# Supplementary material for: CD45-Directed CAR-T Cells with CD45 Knockout Efficiently Kill Myeloid Leukemia and Lymphoma Cells In Vitro Even after Extended Culture
Source: Cancers (Basel). 2024 Jan 12;16(2):334. doi: 10.3390/cancers16020334 (PMC10814116; doi:10.3390/cancers16020334)
Supplement: Supplementary file 1 [file cancers-16-00334-s001.zip › cancers-2773493-supplementary.pdf]

Supplementary Figures & Table

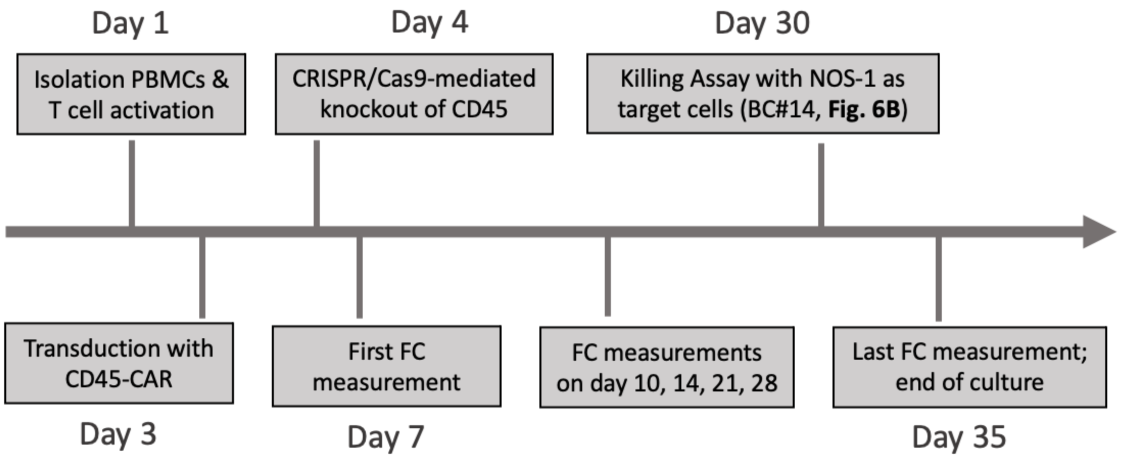

Suppl. Figure S1: Timeline of the experiment with BC#13-#15, CD45^{ko}/CD45CAR-T cells produced following protocol P1.

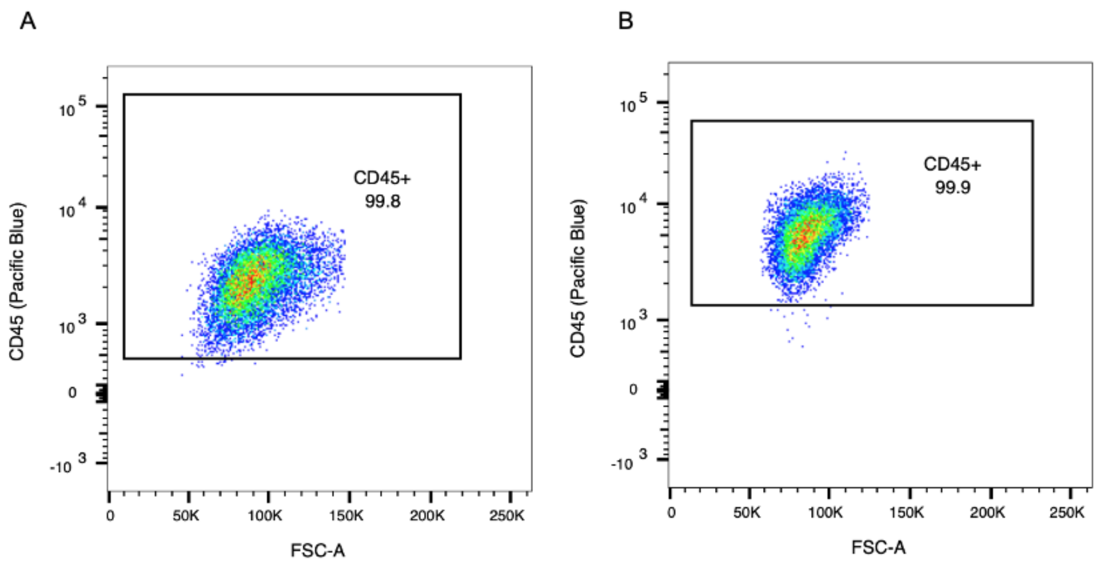

Suppl. Figure S2: Homogenous CD45 expression in (**A**) Ramos-GFP+ and (**B**) NOS-1-GFP + cells. Cells were stained with VioBlue-labelled CD45 antibodies and analyzed by FC.

Suppl. Table S1: Comparison of cell outcomes at day 12 of protocols 1 & 2 for BC#6 (small-scale)

	Cell No [x10^6]	CAR$^+$ [%]	CD45^{ko} [%]	CD45^{ko}/CD45-CAR [x10^6]
P1: Transduction → knockout	1.3	15.6	96.8	0.20
P2: Knockout → transduction	0.95	33.2	66.0	0.21
